# Transcription and Beyond: Delineating FOXG1 Function in Cortical Development and Disorders

**DOI:** 10.3389/fncel.2020.00035

**Published:** 2020-02-25

**Authors:** Pei-Shan Hou, Darren Ó hAilín, Tanja Vogel, Carina Hanashima

**Affiliations:** ^1^Laboratory for Developmental Biology, Department of Biology, Faculty of Education and Integrated Arts and Sciences, Waseda University, Tokyo, Japan; ^2^Institute of Anatomy and Cell Biology, School of Medicine, National Yang-Ming University, Taipei, Taiwan; ^3^Department of Molecular Embryology, Institute of Anatomy and Cell Biology, Medical Faculty, University of Freiburg, Freiburg, Germany; ^4^Center for Basics in NeuroModulation (NeuroModul Basics), Faculty of Medicine, University of Freiburg, Freiburg, Germany; ^5^Department of Integrative Bioscience and Biomedical Engineering, Graduate School of Advanced Science and Engineering, Waseda University Center for Advanced Biomedical Sciences, Tokyo, Japan

**Keywords:** *FOXG1*, cortical development, FOXG1 syndrome, transcription factor, posttranscriptional regulation, cellular reprogramming, cortical organoid

## Abstract

Forkhead Box G1 (*FOXG1*) is a member of the Forkhead family of genes with non-redundant roles in brain development, where alteration of this gene’s expression significantly affects the formation and function of the mammalian cerebral cortex. *FOXG1* haploinsufficiency in humans is associated with prominent differences in brain size and impaired intellectual development noticeable in early childhood, while homozygous mutations are typically fatal. As such, *FOXG1* has been implicated in a wide spectrum of congenital brain disorders, including the congenital variant of Rett syndrome, infantile spasms, microcephaly, autism spectrum disorder (ASD) and schizophrenia. Recent technological advances have yielded greater insight into phenotypic variations observed in FOXG1 syndrome, molecular mechanisms underlying pathogenesis of the disease, and multifaceted roles of *FOXG1* expression. In this review, we explore the emerging mechanisms of *FOXG1* in a range of transcriptional to posttranscriptional events in order to evolve our current view of how a single transcription factor governs the assembly of an elaborate cortical circuit responsible for higher cognitive functions and neurological disorders.

## Introduction

Forkhead Box G1 (FOXG1) is a winged-helix transcription factor that serves as a master regulator for brain development. Among the 44 forkhead family genes identified (Hannenhalli and Kaestner, 2009), *FOXG1* represents a single subclass that is uniquely expressed in the cerebrum and it serves non-redundant roles in cortical development (Tao and Lai, 1992; Xuan et al., 1995), where alteration in its expression severely impacts brain formation and higher cognitive functions. Over recent years, accumulating studies have unveiled the pleiotropic functions of FOXG1 ranging from stem cell proliferation to cortical circuit specialization, and furthermore associated these pathways with human brain disorders. At the molecular level, mechanisms underlying FOXG1 functional diversification involves global transcriptional regulation through cis-regulatory elements in its target genes, as well as fine-tuning of FOXG1 activity at post-transcriptional levels through biochemical and epigenetic processes. In reflection of its functional importance, the non-redundant role of *FOXG1* renders this gene highly vulnerable to subtle mutations introduced in its coding and non-coding sequences, which result in significant changes in brain size, circuit formation, sensorimotor processing, and cognitive behaviors. The aim of this review is to explore the multitude of ways in which FOXG1 mediates diverse developmental and neurological processes under physiological and pathological conditions.

## Cortical Development and FOXG1 Syndrome

FOXG1 was first identified through screening for a Hepatocyte Nuclear Factor 3 (HNF-3/FOXA) homolog expressed in the developing central nervous system (CNS) and was originally named as Brain Factor-1 (BF-1) due to its unique expression in the developing rat telencephalon (Tao and Lai, 1992). *FOXG1* encodes a transcription factor that contains a highly conserved forkhead binding domain and represents the sole member of the FOXG subclass out of the 44 forkhead box family members (Golson and Kaestner, 2016). A growing body of studies over the years have established FOXG1 as both a master regulator of brain development and a key determinant of multiple human brain disorders. Due to its unique and non-redundant expression in the developing cerebrum, alteration of FOXG1 levels highly impacts mammalian brain assembly, where the loss of the gene in mice results in severe microcephaly and mortality at birth (Xuan et al., 1995). The primary function of FOXG1 in brain development would later be elucidated with the generation of conditional knockout mouse lines and genome editing techniques. Detailed analysis revealed pleiotropic roles of FOXG1 in controlling cell proliferation (Hanashima et al., 2002), regional patterning (Hanashima et al., 2007), cell migration (Miyoshi and Fishell, 2012), and circuit assembly (Hanashima et al., 2002, 2004; Kumamoto et al., 2013; Toma et al., 2014; Cargnin et al., 2018; Hou et al., 2019). Applications of genome-wide approaches including transcriptomics and chromatin immunoprecipitation in these studies identified functional targets and direct binding sites of FOXG1, which allowed for the unearthing of parallel downstream neurodevelopmental events through distinct programs.

In humans, the first description of *FOXG1* mutation was reported in a 7-year-old patient with a balanced *de novo* translocation t(2;14)(p22;q12), in which the breakpoint on chromosome 14 disrupts the *FOXG1* transcript (Shoichet et al., 2005). In this case, haploinsufficiency of *FOXG1* was associated with microcephaly, complete agenesis of the corpus callosum, and cognitive disability. These findings highlighted how the loss of one functional copy of *FOXG1* could affect brain development in humans. A phenotypic overlap led to subsequent reports associating *FOXG1* mutation with Rett syndrome, a genetic disorder with early onset of neurological symptoms, as both conditions presented with microcephaly, epileptic seizure, hyperkinetic movement, impaired sleep patterns and intellectual disability (Ariani et al., 2008; Mencarelli et al., 2010; Le Guen et al., 2011). Patients with no apparent changes in the X-linked methyl-CpG binding protein* (MECP2)* gene, which represents 95% of Rett syndrome patients, were subject to genetic analysis and identified *FOXG1* as the gene responsible for the congenital variant of Rett syndrome. Although Rett and FOXG1 syndromes share phenotypic similarities, notable features distinguished the *FOXG1* congenital variant phenotype from the most frequent *MECP2* mutation. In particular, significant differences between the two genes were found in ambulation, receptive language, reciprocity, and sleep, with *FOXG1* subjects exhibiting more severe disability (Ma et al., 2016). In addition to these criteria, neuroimaging techniques have identified characteristic features of the *FOXG1-*mediated disorder, including agenesis of the corpus callosum, blunted gyrification, and reduction in white matter volume in some cases. The significant phenotypical differences between the neurological disorder with underlying *FOXG1* mutations and *MECP2*-associated Rett syndrome led to the designation of FOXG1 syndrome as a distinct disorder.

In addition to Rett syndrome, genetic analysis of *FOXG1* mutations and MRI studies in patients unveiled direct associations between FOXG1 and congenital neurological disorders including autism spectrum disorders (ASD), microcephaly, infantile spasm, and sensory processing disorders. At the genomic level, patients presenting with FOXG1 syndrome display heterozygous variants that harbor *de novo* mutations ranging from truncating, frameshift, nonsense, missense mutations, to duplications in the 14q12 *FOXG1* gene locus (Yeung et al., 2009; Brunetti-Pierri et al., 2011; Seltzer et al., 2014). Such a genetic spectrum of *FOXG1* mutations was broadened by two parallel analyses that employed sequencing of a large cohort of patients with *FOXG1* variants (Mitter et al., 2018; Vegas et al., 2018). Mitter et al. (2018) studied 83 patients that included 54 variants and identified 20 frameshift mutations (37%), 17 missense mutations (31%), 15 nonsense mutations (28%), and 2 in-frame mutations (4%). Vegas et al. (2018) reported 37 *FOXG1* heterozygous mutations including 18 novel mutations with 32 small intragenic mutations and five large deletions in the *FOXG1* gene locus. In this study, a similar frequency of respective mutations was observed: four frameshift (44%), 12 missense (38%), and a minor population of nonsense (4; 13%), and in-frame mutations (2; 6%), indicating the overall contribution of *FOXG1* coding region mutations to neurological symptoms. In alignment with variations in mutations, MRI features have also revealed various patterns of gyrification ranging from pachygyria to normal gyration, partial to complete agenesis of the corpus callosum, and myelination delay. The most severe clinical and MRI anomalies associated with frameshift and nonsense mutations in the N-terminus and forkhead binding domain, whereas milder phenotypes accompanied missense variants in the forkhead binding domain and deletions in the FOXG1-regulatory region (Kortüm et al., 2011; Allou et al., 2012). The most significant phenotypic variability between patients presenting with FOXG1 syndrome appeared in motor and speech development. In contrast, subtle differences were observed in corpus callosum agenesis, delayed myelination, and microcephaly, rendering these three features thus as core FOXG1 syndrome phenotypes.

It is conceivable that the phenotypic variability in FOXG1 syndrome is a consequence of the functional variability of the residual *FOXG1* gene product. Therefore, correlating the location of the mutation within the gene with specific clinical features might lead to a better understanding of the molecular reasons for the heterogeneity amongst FOXG1 syndrome patients. This notion is corroborated by the finding that the amino acid sequence for the forkhead binding domain is highly conserved among vertebrates (Kumamoto and Hanashima, 2017). In contrast, the N-terminal domain of FOXG1 in non-mammalian vertebrates including chicken is largely truncated. Because the C-terminal domain is indispensable for antagonizing Transforming Growth Factor β (TGFβ)-pathway (Dou et al., 2000), these genetic and biochemical studies indicate vertebrate-conserved and mammalian-unique mechanisms of FOXG1 regulation through distinct structural domains, specially encoded in the N-terminus. These and other observations now lead further research unveiling the molecular origin underlying heterogeneity of FOXG1 symptoms at both the genetic and functional level. In the following sections, we further discuss the extent to which respective FOXG1 phenotypes relate to specific molecular programs at distinct regulatory levels.

## From Microcephaly to Glioblastoma: Regulation and Dysregulation of Cell Cycle by FOXG1

### Roles of FOXG1 in Cortical Stem Cell Expansion

In the mammalian CNS, the disproportionate expansion of the cerebrum relative to other structures requires a driving force for cortical growth to generate high numbers of cells, synapses, and overall brain volume. In this regard, FOXG1 plays pivotal roles in controlling the cell cycle to meet the growing demands of the developing cerebral cortex. The first implication of FOXG1 in regulating cortical expansion was unveiled in the phenotype of a constitutive *Foxg1* knockout mouse model that presented severe hypoplasia of the cerebral cortex (Xuan et al., 1995). Genetic and molecular analysis revealed that FOXG1 plays an essential role in regulating neural stem/progenitor cell proliferation and suppressing premature neuronal differentiation through the regulation of the cell cycle (Xuan et al., 1995; Martynoga et al., 2005). Upon loss of FOXG1, cortical stem cells exhibit early lengthening of the cell cycle and increased occurrence of cell cycle exit (Xuan et al., 1995; Hanashima et al., 2002). Replacement of the endogenous *Foxg1* gene with a form bereft of DNA binding further uncovered a distinct requirement for FOXG1 in cell cycle control (Dou et al., 2000; Hanashima et al., 2002). In these cases, replacing two key amino acids within the forkhead domain, asparagine and histidine, with alanines (NH->AA mutant) was sufficient to restore normal cell cycle length, while precocious cell cycle exit was not rescued. These results indicated that the roles of FOXG1 in cell cycle control are regulated by both DNA-binding-dependent and DNA-binding-independent mechanisms (Hanashima et al., 2002).

**Figure 1:**
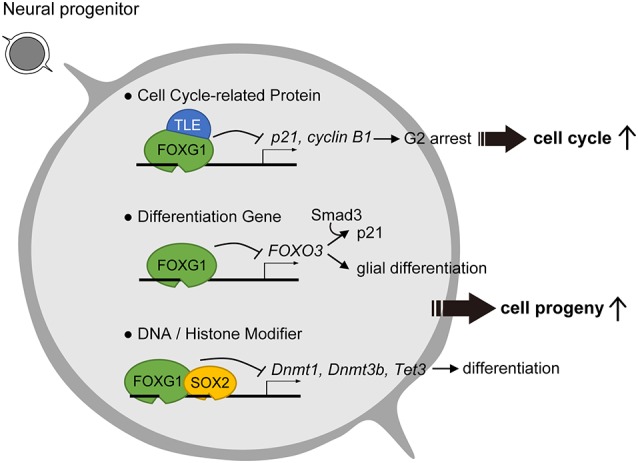
Roles of Forkhead Box G1 (FOXG1) in progenitor cell maintenance and suppression of differentiation. To prevent cell cycle exit, FOXG1 cooperates with Groucho/Transducin-like Enhancer of split (TLE) proteins to inhibit key cell cycle protein expression. FOXG1 also inhibits differentiation gene and DNA/Histone modifiers to prevent neuronal differentiation.

Further investigation of the molecular underpinnings in FOXG1-mediated cell cycle regulation has focused on its roles in antagonizing major cell cycle pathway components. One identified mechanism of FOXG1 is its ability to antagonize the FOXO/SMAD (Sma- and Mad-related protein) pathway, activity of which fosters cortical neuron differentiation (Seoane et al., 2004; Vezzali et al., 2016). In this scheme, FOXG1 inhibits FOXO/SMAD complexes through competition for the consensus forkhead box binding site or through direct association with the FOXO/SMAD complex (Figure 1, middle row). These antagonistic mechanisms reduce the expression of cyclin-dependent kinase inhibitor 1A (*Cdkn1a/p21*). Low levels of CDKN1A prevent cell cycle exit of neural stem cells and promote stem cell pool expansion, thereby enabling the prolonged proliferation of FOXG1-expressing cells.

While the significant loss of brain volume in homozygous *Foxg1* null mutant mice revealed essential requirements for FOXG1 in cortical cell proliferation through cell cycle regulators, understanding its role in regulating cortical cell expansion in human heterozygous mutations requires additional experiments. Indeed, the majority of patients carrying mutations in the *FOXG1* gene exhibit congenital microcephaly at infancy, despite the presence of one functional *FOXG1* allele. These observations strengthen the importance of the *FOXG1* gene dosage in regulating cortical size.

In this regard, studies examining the effect of haploinsufficiency in the developing mouse cortex have elucidated both cell cycle-dependent and -independent mechanisms of FOXG1 in expanding cortical size. As in humans, *Foxg1* heterozygote mice exhibit a significant reduction in the size of the cerebral hemisphere (Eagleson et al., 2007), which becomes readily apparent after the mid-corticogenesis period (embryonic day: E15.5; Siegenthaler et al., 2008). The reduced FOXG1 protein levels in heterozygotes were accompanied by a decrease in Mki67—and Eomesodermin (EOMES/TBR2)—expressing cells, as well as increased CDKN1A expression at early corticogenesis stage (E13.5-E16.5) and cell cycle lengthening at late corticogenesis stage (E17.5; Siegenthaler et al., 2008). Given that *Cdkn1a* is also the target of the TGFβ pathway, loss of one *Foxg1* allele may interfere with its antagonistic effect on the TGFβ pathway, leading to a reduced number of TBR2-expressing intermediate progenitor cells.

### Regulation of Core Cell Cycle and Methyltransferase Gene Expression by FOXG1

Although the key requirement for FOXG1 in cortical cell proliferation has been described, uncovering the downstream targets of FOXG1 and its mode of cell cycle regulation required additional biochemical analysis. It has been reported that the activity of FOXG1 is in part mediated by its association with the Groucho/Transducin-like Enhancer of split (TLE) proteins to form a transcriptional repressor complex (Yao et al., 2001; Marçal et al., 2005). TLE family members contain a C-terminal WD40 repeat domain that associates with the FOXG1 protein, and together they act as a transcriptional corepressor. Interestingly, a distant WD40 containing family member, Gro/TLE-Related Gene Product 6 (GRG6), interferes with FOXG1/TLE-mediated transcriptional repression by preventing the association of FOXG1 and TLE1. TLE1 and GRG6 thus associate with FOXG1 and have opposing roles in stem cell proliferation. Whereas TLE1/FOXG1 overexpression keeps stem cells in a proliferative state, GRG6/FOXG1 has proneural, differentiative functions (Marçal et al., 2005).

Studies into the means by which FOXG1 regulates the cell cycle used key markers CDKN1A and CCNB1/cyclin B1 and demonstrated that overexpression of FOXG1 repressed both *CDKN1A* and *cyclin B1* expression and decreased the proportion of cells in G2 phase (Figure 1; Wang et al., 2018). The knockdown of *FOXG1*, in turn, had the opposite effect. These results suggest that the shortening of the G2/M arrest *via* repression of *CDKN1A* and *cyclin B1* primarily accounts for the accelerated cell cycle and proliferation in the presence of FOXG1 (Figure 1; Wang et al., 2018).

In addition to cell cycle regulators, genome-wide analysis to identify global FOXG1 targets in neural stem cell lines revealed 6897 binding sites with significant enrichment in the canonical forkhead motif (Bulstrode et al., 2017). Interestingly, motif analysis also revealed other neural-development-associated transcription factor binding sites including the basic Helix-Loop-Helix (bHLH), high mobility group (HMG) box, and CAAT box-binding Transcription Factor/Nuclear Factor-1 (CTF/NF1) factors, which are the key components of the neural stem cell transcriptional regulatory network (Mateo et al., 2015). These motifs were for example enriched in genes with methyltransferase function, and concordantly overexpression of FOXG1 affected key regulators of DNA methylation to facilitate dedifferentiation to neural stem cell-like properties (Bulstrode et al., 2017).

### Transcription Factor Network and Epigenome Remodeling in Glioblastoma

While prolonged cell proliferation and expansion of neural stem cells achieved through cell cycle maintenance by FOXG1 are central to the acquisition of an enlarged brain in mammals, aberrant FOXG1 expression equips malignant cells with the capacity for greater self-renewal. It is notable that the chicken ortholog of *FOXG1*, c*-qin* (also known as *CBF-1*), was originally cloned as the cellular homolog of the viral oncogene v*-qin*, which carries transforming activity of the avian sarcoma virus 31 (Chang et al., 1995; Li et al., 1997). Excess of *qin* expression induces overgrowth in the developing chicken neural tube, indicating its conserved roles in expanding the progenitor cell number during brain development (Ahlgren et al., 2003). In humans, augmented *FOXG1* expression has been associated with cancer, where increased *FOXG1* levels have been reported in multiple cancer cell lines and patient tissues (Adesina et al., 2007; Chan et al., 2009; Li et al., 2013).

Glioblastoma, first described by Rudolf Virchow in 1863, is a highly aggressive brain tumor and is considered to be the most lethal primary brain tumor responsible for around 16% of all brain tumors and up to 75% of astrocytic tumors (Urbańska et al., 2014). An increasing body of research has revealed both transcriptional and epigenetic mechanisms that control the maintenance of neural stem cells during homeostasis and perturbation (Patel et al., 2014; Suvà et al., 2014). In this regard, FOX and SRY-box Transcription Factor (SOX) family genes are critical regulators of neural stem cell self-renewal and differentiation. In particular, *FOXG1* is one of the most consistently upregulated genes in glioblastoma-derived neural stem cells (Engström et al., 2012), and the survival of patients is inversely correlated with *FOXG1* mRNA levels in primary tumors (Verginelli et al., 2013). Conversely, knockdown of *FOXG1* by shRNA reduces the proliferation of glioblastoma stem cells (Verginelli et al., 2013) indicating a direct involvement of FOXG1 in the progression of glioblastoma.

Biochemical analysis in glioblastoma cell lines revealed that one function of FOXG1 is to antagonize the effects of TGFβ signaling through its binding to FOXO/SMAD complexes (Figure 1, middle row; Seoane et al., 2004). Assay for transposase-accessible chromatin (ATAC) sequencing to assess genome-wide chromatin accessibility changes between human glioblastoma and control neural stem cells revealed that high FOXG1 level in glioblastoma cells contributes to a modified chromatin landscape (Buenrostro et al., 2013). Unsupervised clustering identified that glioblastoma-derived neural stem cells exhibited a greater diversity in chromatin profile and enriched forkhead box and HMG box motifs, which are bound by the FOX and SOX factors, respectively. Thus, increased FOXG1 protein level and a FOX/SOX-enriched open chromatin profile are features of glioblastoma cells. Concomitant elevated FOXG1 and SOX2 expression levels in glioblastoma enforces neural stem cell identity through transcriptional control of cell cycle and epigenetic regulators including *Foxo3*, Polo Like Kinase 1 (*Plk1*), MYCN Proto-Oncogene (*Mycn*), DNA Methyltransferase 1 (*Dnmt1*), *Dnmt3b*, and Tet Methylcytosine Dioxygenase 3 (*Tet3*; Figure 1, bottom row; Bulstrode et al., 2017). Taken together, FOXG1 and SOX2 act to enhance self-renewal in glioblastoma stem cells through the control of the core cell cycle and epigenetic regulators. As FOXG1 is also observed to be dysregulated in various types of cancer including hepatoblastoma, medulloblastoma, breast cancer, and ovarian cancer (Adesina et al., 2007; Chan et al., 2009; Li et al., 2013), it is plausible that aberrant regulation of FOXG1 expression outside of the nervous system also triggers dysregulated cell cycle and tumorigenesis in pathological conditions.

In addition to the downstream pathways, FOXG1 overexpression in glioblastoma revealed a critical role of Epidermal Growth Factor Receptor (EGFR) in FOXG1 expression control. Changes in EGFR expression due to copy number aberrations and mutations in the *EGFR* gene are among the features of glioblastoma. Epigenome and transcriptome analyses using cell lines and pathological samples have shown that *EGFR* mutations remodel the activated enhancer landscape in glioblastoma, which promotes a more aggressive tumor phenotype through FOXG1- and SOX9-dependent transcriptional regulatory networks (Liu et al., 2015). These studies strengthened the importance of transcriptional and epigenetic remodeling in glioblastoma pathogenesis. Integrating H3K4me1 and H3K27ac ChIP-seq analysis and RNA-seq further revealed significantly enriched enhancers near the oncogenic EGFR mutant (EGFRvIII) upregulated transcripts, with these regions harboring binding motifs for the FOX and SOX family transcription factors. Expression of EGFRvIII in glioblastoma cells also increased SOX9 and FOXG1 mRNA and protein levels (Liu et al., 2015), and thus the increased expression of both transcription factors in EGFR-mediated epigenetic remodeling is a key regulatory network in triggering dedifferentiation to a neural stem cell state.

Notably, FOXG1 and SOX2 are also key cellular reprogramming factors that coordinate in the direct reprogramming of fibroblasts to neural stem cells (Lujan et al., 2012). Thus, it is plausible that high levels of FOXG1 in glioblastoma suppress differentiation and trigger dedifferentiation through reprogramming to a neural-stem-cell-like state. This could render cells more vulnerable to malignant transformation since neural stem cells require a less complex stochastic mutation program than astrocytes in initiating gliomagenesis (Jacques et al., 2010; Vitucci et al., 2017). Further insight into the function of FOXG1 in glioma pathogenesis and progression would greatly improve our understanding of glioblastoma as well as brain-specific features of stem cell regulation.

## Spatiotemporal Dynamics of FOXG1 in Creating Cortical Cell Diversity

### FOXG1 Instructs Cortical Laminar Subtypes

In parallel to expanding the progenitor cell pool through control of cell cycle regulators, the onset of FOXG1 expression in the developing forebrain triggers a cascade of genetic and molecular events in corticogenesis. These events include dorsoventral patterning of the telencephalon to designate future neocortex and basal ganglia compartments and specifying cell types through global switches in gene expression of downstream target genes. The induction of *FOXG1* and early patterning of the forebrain appear to be largely conserved among vertebrates, where compartmentalization of the forebrain is established by reciprocal actions between morphogens and transcription factors. In the future telencephalic territory, Sine Oculis-related Homeobox 3 (*Six3*) expressed in the anterior neural plate (Oliver et al., 1995) establishes the competence for *Foxg1* induction, whereby Fibroblast Growth Factor 8 (*FGF8*) expressed in the anterior neural ridge induces *Foxg1* and organizes the telencephalic region (Oliver et al., 1995; Suda et al., 1997; Lagutin et al., 2003). Notably, while FOXG1 serves as a hallmark of the telencephalon in vertebrates (Toresson et al., 1998), FGF also induces the expression of FOXG in the *Saccoglossus kowalevskii* proboscis (Pani et al., 2012), implying that the expression of FOXG1 played important roles in anterior ectoderm patterning during evolution. In vertebrates, however, FOXG1 is also regulated by FGF8 through Dickkopf WNT Signaling Pathway Inhibitor (DKK), which provides a positive feedback regulation of FGF signaling (Aguiar et al., 2014). The stepwise regulation and subsequent prolonging of FOXG1 expression may have augmented the proliferation and expansion of the telencephalon in the vertebrate lineage. Following its onset, FOXG1 is highly expressed in the telencephalon and its derivative structures including the cerebral cortex, caudate-putamen, hippocampus, retina and sensory placodes (Tao and Lai, 1992).

**Figure 2:**
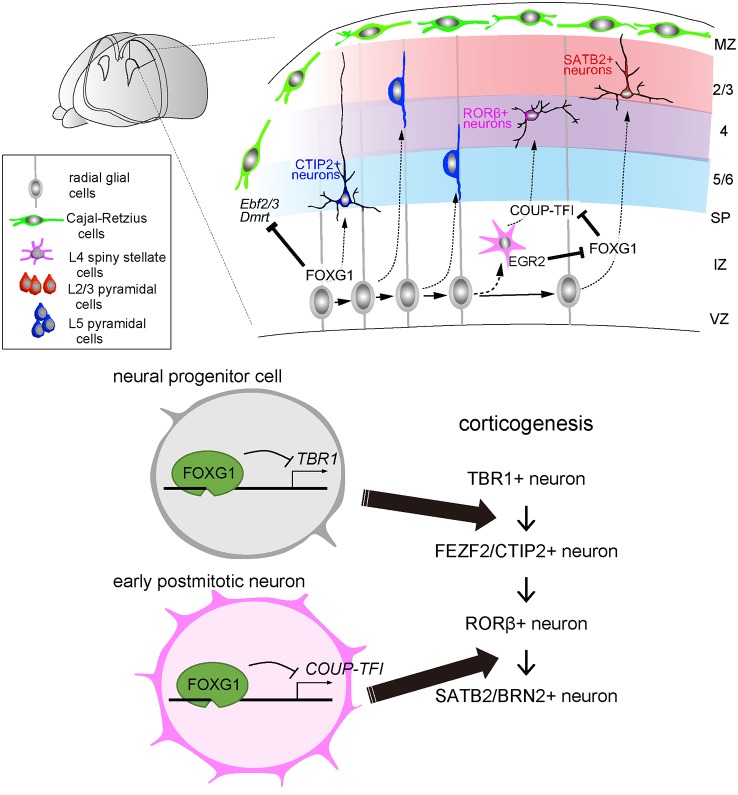
FOXG1 directs multiple laminar fate decisions in the cerebral cortex. During corticogenesis, cortical neurons are sequentially produced, migrate and integrate into the destined layers. In this process, the onset of FOXG1 in the progenitor cells suppresses the production of L1/6 TBR1-expressing neurons and switches to L5 FEZF2/CTIP2-expressing neuron production. Later in early postmitotic neurons, FOXG1 inhibits COUP-TFI to switch the neuronal production from L4 Related Orphan Receptor B (RORβ)—to L2/3 SATB2/BRN2—expressing neurons.

After the major compartments of the telencephalon are established, FOXG1 expression orchestrates the sequential specification of projection neuron subtypes in the dorsal telencephalon (Figure 2). Cortical progenitor cells undergo asymmetric division and begin producing T-Box Brain Transcription Factor 1 (TBR1)-expressing neurons, which become layer 1 and layer 6 neurons at the surface and the deepest regions of the cortical plate. Progenitor cells further produce layer 5 FEZ Family Zinc Finger 2 (FEZF2)- and BAF Chromatin Remodeling Complex Subunit BCL11B (BCL11B/CTIP2)-expressing corticospinal projection cells, followed by layer 4 RAR Related Orphan Receptor B (RORβ)-expressing sensory input cells, and then layer 2/3 Special AT-Rich Sequence-Binding Protein 2 (SATB2) and POU Class 3 Homeobox 2 (POU3F2/BRN2)-expressing callosal projection neurons. These neurons integrate into the cortical plate following an inside-out lamination pattern, in which later-born neurons migrate past earlier-born neurons and take a position at the superficial region. Notably, while FOXG1 is expressed in many of the cortical progenitor cells and neurons, its function in progenitor cell proliferation and neuronal differentiation differs between subtypes and varies in a spatiotemporal manner (Hanashima et al., 2004; Toma et al., 2014; Hou et al., 2019). The onset of FOXG1 expression in cortical progenitor cells terminates the production of the earliest born neurons, i.e., Cajal-Retzius cells, through direct inhibition of a default transcriptional network. This network includes, as revealed by transcriptome and FOXG1-chromatin-immunoprecipitation (ChIP)-sequencing, *Tbr1*, Doublesex- And Mab-3-Related Transcription Factor A1 (*Dmrta1*), Early B Cell Factor 2 (*Ebf2*), and *Ebf3* (Hanashima et al., 2004, 2007; Kumamoto et al., 2013). This transcription factor network, in turn, switches cortical neurogenesis to layer 5 FEZF2- and CTIP2-expressing neuron production (Srinivasan et al., 2012; Toma et al., 2014; Figure 2, bottom panel). After triggering deep-layer projection neuron production, FOXG1 expression is maintained in cortical progenitor cells, while its expression becomes variable in postmitotic neurons, both in the intermediate zone and after entering the cortical plate. While the activation of FOXG1 controls cortical plate entry (Miyoshi and Fishell, 2012), a recent study demonstrates that its expression during mid- and late-corticogenesis in the intermediate zone is critical for segregating later-born subtypes of cortical neurons (Hou et al., 2019). The timely downregulation of FOXG1 by Early Growth Response 2 (EGR2), a TGFβ downstream target, in the lower intermediate zone where cells have just exited the cell cycle causes derepression of Nuclear Receptor Subfamily 2 Group F Member 1/Chicken Ovalbumin Upstream Promoter-Transcription Factor I* (Nr2f1/COUP-TFI)*, triggering layer 4 cell competence. By contrast, removal of EGR2 target sites elevates *Foxg1* expression and promotes the acquisition of SATB2/BRN2-positive callosal projection neuron fate (Hou et al., 2019; Figure 2, bottom panel). As *FOXG1* haploinsufficiency results in agenesis of the corpus callosum in humans (Shoichet et al., 2005) and mice (Cargnin et al., 2018) due to the defect in development of upper-layer projection neurons (Siegenthaler et al., 2008), together these studies imply that two functional copies of the *Foxg1* gene are required to control cortical neuron production and axon development for cortical circuit formation characteristic of FOXG1 disorders. Collectively, multiple functions of FOXG1 at distinct developmental stages highlight its comprehensive roles in establishing the elaborate cortical circuits through selective targeting of downstream genes in a temporally coordinated manner.

### FOXG1 Balances Excitatory and Inhibitory Inputs in the Neocortical Circuit

While the requirement for FOXG1 in the determination of cortical glutamatergic subtypes has been well delineated through conditional loss-of-function experiments (Figure 2), the impact of FOXG1 on GABAergic inhibitory subtypes have remained less clear due to their higher susceptibility to loss of FOXG1 during early development (Manuel et al., 2010). Ventral telencephalic progenitor cells express higher levels of FOXG1 as compared with dorsal progenitors (Danesin et al., 2009; Danesin and Houart, 2012), and *Foxg1* knockout cells cannot contribute to ventral telencephalic cells that express NK2 Homeobox 1 (*Nkx2-1*), Achaete-scute Family bHLH Transcription Factor 1 (*Ascl1*/*Mash1*), or GS Homeobox 2 (Gsx2/*Gsh2*, Martynoga et al., 2005). The expression differences in FOXG1 between the dorsal and ventral telencephalic progenitor cells is critical for the establishment of excitatory and inhibitory subtypes in the mature cortex, as changes in this balance underlies phenotypical variations ranging from epileptic seizure to ASD, both of which are influenced by *FOXG1* expression levels. For instance, neurons differentiated from patient-derived induced pluripotent stem cells express higher levels of inhibitory synaptic marker proteins (Mariani et al., 2015). Imbalanced synaptic inputs towards inhibitory signals seem not to be restricted to FOXG1 syndrome but have been observed in other ASD including typical and atypical Rett syndrome (Patriarchi et al., 2016). Moreover, transcriptome analyses using cortical organoids derived from ASD patients have revealed changes in transcripts involved in cell proliferation, neuronal differentiation and connectivity, featuring accelerated cell cycle and overproduction of GABAergic neurons (Mariani et al., 2015). A critical dysregulation of FOXG1 in the ASD cortex could be inferred as increased *FOXG1* transcription despite the absence of a gene mutation. FOXG1 influence on the development of inhibitory neurons was further evaluated by suppression of *FOXG1* using shRNA, which was sufficient to reduce the over-proliferation of GABAergic progenitor cells, indicating that the bias toward GABAergic neurogenesis by dysregulated FOXG1 expression is a primary mediator of ASD. These results indicate that fine-tuned FOXG1 levels are essential for balancing neurotransmitter subtypes for optimized cortical circuit transmission. Further exploring the upstream mechanisms of FOXG1 expression augmentation in ASD-derived organoids, as well as its active manipulation as discussed in the later section, will advance our understanding of the pathology in ASD.

### FOXG1 Affects Cells in Non-neuronal Lineages

While the regulatory network by which FOXG1 mediates cortical neuron subtype-specification has been described, its function in instructing non-neuronal cell lineages of the cerebral cortex remains less understood. It has been shown that overexpression of FOXG1 in cultured neural precursors increases the proportion of neural stem cells at the expense of glial progenitor cells (Brancaccio et al., 2010). These findings are consistent with the described role of FOXG1 as one of the key regulators for neuronal reprogramming. Forced expression of FOXG1 in non-neural cells (Lujan et al., 2012; Raciti et al., 2013; Colasante et al., 2015) and mouse astrocytes (Ma et al., 2018) is sufficient to drive neurogenic competence, as discussed later in the section for *cellular reprogramming*.

Interestingly, the aforementioned opposing actions between FOXG1 and COUP-TFI transcription factors in defining temporal layer identity also appear applicable to neurogenic-gliogenic temporal fate switch decisions. *In vitro* knockdown of *COUP-TFI/II* in embryoid-body-derived neural stem/progenitor cells results in a significantly increased proportion of neurogenic cells, which is accompanied with increased Histone H3 Lysine 9 Dimethylation (H3K9me2) and decreased H3K4me2 as well as Histone H3 acetylation at the Signal Transducer and Activator of Transcription 3 (Stat3)-binding site of the Glial Fibrillary Acidic Protein (*Gfap*) promoter (Naka et al., 2008). Furthermore, the introduction of a *COUP-TFI/II* knockdown lentivirus in the early embryonic mouse brain results in neuronal expansion at the expense of GFAP-expressing astrocytes and SOX10- and Oligodendrocyte Transcription Factor 2 (OLIG2)-expressing oligodendrocyte precursor cells (Naka et al., 2008). Together with the roles of FOXG1 in driving neurogenic potency, these experiments indicate the reciprocal actions between FOXG1 and COUP-TFI in establishing neuronal and gliogenic competence in the developing cerebral cortex.

Notably, while FOXG1 predominantly instructs neurogenic lineage *in vitro*, the temporal window of neurogenic competence appears to be regulated independently of *Foxg1* expression. In *Foxg1* constitutive knockout mice, progenitor cells give rise to Cajal-Retzius neurons (Hanashima et al., 2004), whereas delayed onset of FOXG1 expression *in vivo* using a tetracycline (tet)-inducible system is sufficient to shift the deep- and upper-layer projection neuron production window, but is not sufficient to override the gliogenic competence (Toma et al., 2014). Furthermore, a recent study has indicated that FOXG1 may play additional roles within the gliogenic lineage. Single-cell RNA-seq revealed two molecularly distinct astrocyte populations, expressing markers including GFAP and Milk Fat Globule-EGF Factor 8 (MFGE8) in the cerebral cortex (Zeisel et al., 2015). Using FOXG1-Cre-dependent recombination *in vitro* and *in vivo*, suppression of TGFβ signaling by floxed*Tgfbr2* alleles revealed that while GFAP-expressing astrocytes from both dorsal and ventral telencephalon derivatives were affected, only dorsally derived MFGE8-expressing astrocytes were affected upon loss of *Tgfbr2* (Weise et al., 2018). This points to the presence of FOXG1-negative astrocytic progenitor cells that maintain the TGFBR2 receptor upon FOXG1-Cre mediated recombination, revealing distinct molecular subtypes of astrocytes segregated into a FOXG1 and non-FOXG1 lineage. These fate-mapping experiments imply FOXG1 expression during early brain development may serve as a hallmark of astrocyte heterogeneity.

## Beyond the Nucleus: Posttranscriptional and Cytosolic Roles of FOXG1 in Corticogenesis

### Dynamic Localization of FOXG1 Regulates Neuronal Differentiation

Extensive studies using genetic and biochemical analyses have revealed downstream transcriptional targets of FOXG1 and how they regulate multiple steps of cortical development. In contrast, the upstream mechanisms by which FOXG1 expression and its activity is regulated has remained less understood. In this regard, studies have demonstrated that chromatin remodeling protein SMARCA1/SNF2L (SWI/SNF Related Matrix Associated Actin-dependent Regulator of Chromatin) and EGR2 both physically bind to the* Foxg1* gene locus and repress its expression (Yip et al., 2012; Hou et al., 2019). Furthermore, experiments using *Xenopus* and mouse models have revealed a dynamic shuttling of the FOXG1 protein in the embryonic brain. FOXG1 localizes in the nucleus of progenitor cells whereas cytoplasmic localization was observed in differentiating cells (Regad et al., 2007). The subcellular localization of FOXG1 is regulated post-translationally by reciprocal signaling between Casein Kinase I (CKI) and FGF, where CKI phosphorylates FOXG1 at the N-terminus Ser19 site to promote nuclear import. FGF, in turn, promotes FOXG1 nuclear export through phosphorylation of Thr226 within the DNA-binding domain and triggers neuronal differentiation, revealing an antagonistic regulation between CKI and FGF on FOXG1 expression (Regad et al., 2007). These findings uncovered that the dynamics of FOXG1 localization and its regulation at post-translational levels are critical for the activity of the FOXG1 protein in neuronal differentiation.

**Figure 3:**
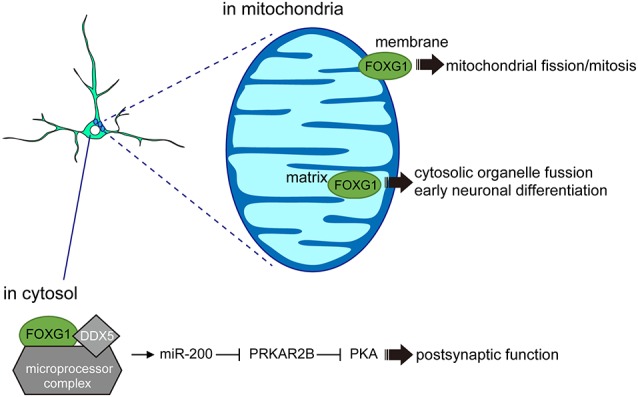
FOXG1 functions outside the nucleus. In the cytosol, FOXG1 cooperates with DDX5 and microprocessors to induce miR-200 biogenesis and promote postsynaptic function. In the mitochondrial membrane, FOXG1 controls mitochondrial mitosis and its expression in the matrix regulates early neuronal differentiation.

A separate study supports the view that FOXG1 also acts outside of the nucleus to regulate neuronal differentiation. Using mouse cortical tissue, primary neurons, and cell lines, a fraction of FOXG1 was shown to localize within the mitochondria (Pancrazi et al., 2015). The specific transport of FOXG1 to the mitochondria appears to be membrane-potential-dependent, where amino acids (aa) 277–302 act as a critical domain for mitochondrial localization (Figure 3). Overexpression of the full-length FOXG1 enhanced mitochondrial membrane potential and promoted mitochondrial fission and mitosis. By contrast, overexpression of the C-terminal fragment of FOXG1 (aa 272–481), which selectively localizes to the mitochondrial matrix, enhanced organelle fusion and promoted the early phase of neuronal differentiation (Pancrazi et al., 2015). These findings imply that the subcellular localization of FOXG1 instructs the mitochondrial function to regulate their replication, bioenergetics, and neuronal differentiation, linking for the first time FOXG1 function to non-nuclear organelles during brain development. Together, these findings indicate that FOXG1 plays roles beyond chromatin-mediated transcriptional regulation. Such FOXG1 function outside of the nucleus also opens a new avenue in its roles to control neuronal maturation or plasticity, where FOXG1 may actively participate in the axonal and dendritic organization, and synaptic formation (Mattson et al., 2008; Gioran et al., 2014).

### Post-transcriptional Regulation *via* miRNA Processing Pathways

Further evidence that FOXG1 does not exclusively act at the chromatin comes from an interactome study which demonstrated that the fraction of FOXG1-interacting proteins in N2a cells (Weise et al., 2019) was enriched for proteins affecting post-transcriptional regulation. For example, FOXG1 associates with the microprocessor complex *via* interaction with DEAD Box Polypeptide 5 (DDX5), demonstrating the role of the FOXG1/DDX5 axis in miRNA biogenesis. Small RNA sequencing comparing hippocampal cells of 6-week-old *Foxg1^cre/+^* heterozygous mice with wildtype littermates revealed a small number of miRNAs that were significantly altered in their expression levels. One of the miRNAs with a reduced expression upon *FOXG1* reduction belongs to the miR-200 family, which is known to control similar processes to those of FOXG1 in the developing forebrain (Pandey et al., 2015; Beclin et al., 2016). Furthermore, *Foxg1* itself has been reported to be one of the miR-200 targets in other model systems (Choi et al., 2008; Garaffo et al., 2015; Zeng et al., 2016), suggesting that *FOXG1* expression levels may also be regulated through a miR-200-dependent feedback loop. RNA-seq following miR-200 overexpression and intersection of this cohort with RNA-seq datasets of the *Foxg1*-heterozygous hippocampus RNA-seq revealed an overlap of 35 potential target genes (Weise et al., 2019). In particular, cAMP-dependent Protein Kinase Type II-Beta Regulatory Subunit (*Prkar2b*) was identified as a common target for miR-200 and FOXG1 in N2a and hippocampal cells. Given that PRKAR2B inhibits postsynaptic functions by attenuating protein kinase A (PKA) activity (Figure 3), the increased PRKAR2B levels observed in *Foxg1* heterozygous mouse models may contribute to neuronal dysfunctions during synaptic transmission. This study identified that FOXG1 affects PRKAR2B expression at multiple levels: through direct transcriptional repression and by targeting *Prkar2b* transcripts through the miR-200 family. Together with the roles of FOXG1 in mitochondria, these findings reveal novel functions of FOXG1 beyond transcriptional regulation, in which interaction between FOXG1, DDX5 and the microprocessor complex controls the maturation of miRNAs.

## Genomic Landscape Underlying FOXG1 Expression in Human Cognitive Function and Psychiatric Disorders

### FOXG1 in Human Cognitive Function and Impairment

While our understanding of FOXG1 has for the most part centered around its developmental roles and implications in early postnatal congenital anomalies, mounting evidence implicates perturbed functions of FOXG1 in neural plasticity and consequential social and cognitive-behavioral defects. Indeed, FOXG1 is expressed in both the neurogenic niche and differentiated neurons of the adult cerebral cortex (Shen et al., 2006)^1^, indicating its roles in cognitive function and neural plasticity. Manipulating the levels of FOXG1 in primary culture affects dendritic growth of differentiating neurons, where FOXG1 overexpression promotes dendritic elongation and neurite branching, which is in part mediated through positive regulation of Hes Family bHLH Transcription Factor 1 (HES1) and cAMP Responsive Element Binding Protein 1 (CREB1; Chiola et al., 2019). These results indicate that FOXG1 may serve active roles in circuit formation. In the adult hippocampus, *Foxg1* haploinsufficiency leads to a progressive decrease in the number of dentate granule cells (Shen et al., 2006). Accompanying this reduction in cell number, cognitive and behavioral deficits including impaired contextual memory symptomatic of compromised hippocampal function were observed. Complete removal of *Foxg1* in adult neurons using an inducible Calcium/Calmodulin-dependent Protein Kinase II alpha (*Camk2α)-Cre^ER^* combined with floxed *Foxg1* mice revealed that *Foxg1* deletion results in deficit in spatial learning and memory as assessed by Morris water maze, as well as a significant reduction in contextual and cued fear conditioning assay (Yu et al., 2019). These features were associated with impaired long-term potentiation and a significant decrease in the amplitude of spontaneous excitatory postsynaptic currents in CA1 pyramidal neurons. Together, these studies lend credence to the position that appropriate level of FOXG1 is required for proper synaptic transmission, uncovering a new function for FOXG1 in controlling neural plasticity in the mature neocortex.

### High Resolution 3D Topological Map Associates FOXG1 Regulation and Psychiatric Disorders

The view of haploinsufficiency as the underlying pathogenetic mechanism of the FOXG1 syndrome phenotype is evidenced by the observed *FOXG1* deletions and point mutations present in the disorder (Shoichet et al., 2005; Redin et al., 2017; Mitter et al., 2018). Key developmental genes are located in evolutionarily conserved genomic landscapes, which are enriched in non-coding elements that can act as regulatory sequences for target genes located up to several hundred kilobases away. In such examples, the formation of chromatin loops brings these long-range regulatory elements into close proximity with the promoter, condensing an array of regulatory elements to drive expression of a target gene in a spatiotemporally coordinated manner. With regards to the *FOXG1* gene, two putative *cis*-regulatory elements of high evolutionary conservation (*hs556* and *hs342)* were originally proposed to be regulators of *FOXG1* expression (Kortüm et al., 2011). However, the deletion of *hs566* was also detected in healthy individuals, raising the forebrain-specific enhancer *hs342* as the first candidate *cis*-regulatory element implicated in FOXG1 syndrome. A subsequent report mapping seven patients with severe encephalopathies revealed five *cis*-acting regulatory elements: one neural tube-specific enhancer and four silencers affecting *FOXG1* at genomic and transcriptional levels (Allou et al., 2012), supporting the presence of long-range regulatory interactions between these regulatory elements and *FOXG1* expression. Mapping of additional translocations with virtual 4C and Hi-C DNA-DNA analysis identified regulatory regions across topologically associated domains (TAD) boundaries associated with different clinical phenotypes (Mehrjouy et al., 2018), providing a genome-wide landscape of *FOXG1* regulators at chromosomal levels.

While the topological organization of *FOXG1* regulatory elements has begun to unveil with the advent of chromosome conformation capture technology, how FOXG1 itself regulates global gene expression in a spatiotemporally coordinated manner remained to be defined. In the developing mouse neocortex, ChIP-seq studies using FOXG1 antibodies have revealed key transcriptional networks responsible for neuronal specification, migration and axonal guidance regulation (Kumamoto et al., 2013; Cargnin et al., 2018). However, the organization of FOXG1 targets at chromosomal levels in human brain development has remained unclear. As three-dimensional physical interactions within chromosomes are responsible for tissue-specific gene expression regulation (Lieberman-Aiden et al., 2009), high-resolution 3D maps of chromatin contacts during human corticogenesis allowed for the discovery of regulatory gene networks and their dysregulation in neurodevelopmental disorders. In this regard, high-resolution screening has associated FOXG1 with critical neuropsychiatric disorders, where construction of Hi-C libraries of the mid-gestation human cerebral cortex from the cortical plate and germinal zone cells revealed schizophrenia-associated regulatory single nucleotide polymorphisms that physically interact with FOXG1 at genomic levels (Won et al., 2016). With *FOXG1* highlighted as a candidate schizophrenia-risk gene involved in its pathogenesis, the authors employed CRISPR/Cas9-mediated genome editing to delete schizophrenia-associated *rs1191551* in primary human neural progenitor cells and found reduced expression of FOXG1 in these cells. As such, regulation of *FOXG1* by this region is likely to occur in human cortical development (Won et al., 2016). These findings establish a plausible link between the acquisition of enhancers involved in cognitive functions and predisposition to neuropsychiatric disorders through physical interactions with major neurodevelopmental genes including *FOXG1*.

## Manipulating FOXG1 for Cellular Reprogramming and in Cortical Organoids

### The Impact of FOXG1 on Cellular Reprogramming

Until recently, therapeutic approaches to treat neurodevelopmental disorders and progressive neurodegenerative diseases have remained a challenge due to the limited understanding of their pathological processes (Bredesen et al., 2006). To overcome this obstacle, effort over the past decade has been directed towards reprogramming cell fate into specific neural lineages to recapitulate developmental or pathological features by manipulating the expression of critical neural transcription factors (Kim et al., 2011; Thier et al., 2012). Given its role in progenitor cells (Figure 1), FOXG1, together with SOX2 and BRN2 are recognized as key reprogramming factors that redirect somatic fibroblasts to induced neural progenitor cells (iNP; Lujan et al., 2012). Among these factors, FOXG1 initiates iNP conversion, SOX2 is required for iNP maturation, and BRN2 is required for iNPs to gain potency for oligodendrocyte differentiation. In addition to SOX2/ BRN2, FOXG1 also participates as a core component for converting somatic fibroblasts into neural progenitors (Raciti et al., 2013; Hou et al., 2017), establishing FOXG1 as a keystone in directing neural fate. These studies also revealed that the potency of iNPs is slightly restricted compared to the earliest embryonic neural progenitor cells by specific transcription factor configurations that include FOXG1 (Hou et al., 2017). This observation is consistent with the earliest developmental onset of FOXG1 expression that restricts the potency of early-born neuron differentiation in developing cortical progenitor cells (Figure 2; Hanashima et al., 2004; Kumamoto et al., 2013).

Taking advantage of FOXG1 in neural fate establishment and its expression and function in interneurons, somatic fibroblasts were reprogrammed into induced GABAergic neurons (iGABA) by combining expression of *FOXG1* with the neurogenic genes *SOX2*, *ASCL1*, and interneuron genes Distal-less Homeobox 5 (*DLX5)* and LIM Homeobox Protein 6 (*LHX6*; Colasante et al., 2015). In line with iNP studies, the ectopic FOXG1 expression initiates the reprogramming process towards the neural state. Subsequently, the presence of FOXG1 expression with pluripotent pioneer factor SOX2 (Soufi et al., 2015) and neuronal fate determinant ASCL1 (Wapinski et al., 2013; Raposo et al., 2015) established the interneuron identity through activation of critical interneuron gene DLX2, suggesting an alternative role of FOXG1 as a co-factor in determining the trajectory of neural cell identity.

Although cell-reprogramming studies establish FOXG1 as a critical factor for directing neuronal identity, the underlying mechanism of action in this function remains obscure. One possibility is that FOXG1 acts as a pioneer factor that targets closed chromatin regions to increase the accessibility of key downstream genes (Iwafuchi-Doi and Zaret, 2014). However, in iGABA, FOXG1 does not directly target the *DLX1/2* enhancer but rather supports SOX1 and ASCL1 to facilitate *DLX1/2* activation (Colasante et al., 2015). In addition, iNP studies also demonstrate that FOXG1 alone is not sufficient to induce neural fate (Lujan et al., 2012; Raciti et al., 2013; Hou et al., 2017). Considering its role in neuronal fate, the current view is that FOXG1 functions as a key suppressor (Hanashima et al., 2004; Kumamoto et al., 2013; Toma et al., 2014; Hou et al., 2019) rather than a pioneer factor. As such, FOXG1 likely serves as a blockade against other fate trajectories in order to secure fate establishment in the development and reprogramming process.

### Manipulating FOXG1 in Cortical Organoid Models

In FOXG1 syndrome patients, deletions or missense mutations on one *FOXG1* allele cause severe neurodevelopmental defects (Florian et al., 2012). Given that FOXG1 syndrome patients maintain at least one functioning *FOXG1* allele, the wide spectrum of phenotypic features observed in FOXG1 syndrome may reflect variability in the expression levels of FOXG1. Indeed, even a subtle reduction in the expression levels of pivotal genes such as *FOXG1* can have a disastrous impact on the developmental process. Given that we cannot directly manipulate these mutations in the developing human brain, cortical organoids derived from pluripotent stem cells serve as a viable substitute as they recapitulate human first-trimester cortical development *in vitro* (Trujillo et al., 2019). Establishing an *in vitro* platform to manipulate and assess FOXG1-mediated gene networks in cultured cortical organoids will allow assessing gene functions and therapeutic avenues in human brain disorders including FOXG1 syndrome. Indeed, recent efforts have successfully established human cortical organoid models that organize into discrete regions of neuronal identity that mimic human organogenesis *in vitro*, and this allowed for more advanced modeling of human developmental disorders (Lancaster et al., 2013; Mariani et al., 2015).

While considerable insight could be gleaned from genetic tools that dampen or completely extinguish specific gene expression, understanding the precise dose-dependent effects of their protein products in pathological conditions caused by haploinsufficiency remained a major hurdle to overcome. In this regard, researchers developed a new approach that combines cortical organoids grown from human pluripotent stem cells (hPSCs) and CRISPR/Cas9 with small molecule-assisted shut-off (SMASh), allowing for endogenous proteins to be targeted and their abundance to be precisely altered. This SMASh technique utilizes proteins fused to a self-removing degron, which allows reversible and dose-dependent shut-off by the administration of small molecules (Chung et al., 2015). In the absence of these molecules, SMASh self-cleaves and protects the target protein against degradation. This process can also be selectively and efficiently blocked by NS3 protease inhibitors, resulting in the degradation of the fused protein. Researchers used this system to assess the relationship between FOXG1 protein dosage and pathological features of FOXG1 syndromes, such as microcephaly and aberrant cortical patterning, revealing that the cellular constitution of human brain organoids exhibited a FOXG1 dose-dependent response (Zhu et al., 2019). Reducing FOXG1 protein to 59.2% of its physiological abundance led to mild defects in GABAergic interneuron development, while a reduction to 28.9% severely impacted the production of medial ganglionic eminence (MGE)—derived interneurons. Notably, the reduction of FOXG1–60% in these organoids did not alter the proportion of Doublecortin (DCX)—to Tubulin beta 3 class III (TUBB3)-expressing cells, but it severely dampened action potential firing, suggesting that such a reduction could potentially lead to dysfunction in brain regions lacking major morphological defects. The SMASh system thus serves as an efficient tool for studying the neuronal differentiation events underlying various neurological symptoms such as epilepsy or seizures on an *in vitro* platform. This opens new avenues of investigation into FOXG1 syndrome pathogenesis and could potentially unearth molecular targets in unexplored FOXG1 pathways for the development of safe therapeutic applications.

## Future Perspectives

Understanding the developmental mechanisms that link individual genes and cell types to the corresponding behavior observed in humans present a fundamental step in the pursuit of functional treatments for FOXG1 disorders. It has until recently, however, remained a challenge to link embryonic gene expression to phenotypic variations observed in the FOXG1 syndrome. The difficulty in dissecting the direct and indirect events leading to the observed changes upon a single gene mutation presented a major roadblock in these endeavors, as did the paucity of means to assess the dose-dependent function of FOXG1 in developing embryos through precise control of protein levels in targeted cell types. This review article considers the progress over recent years that integrates complementary *in vivo* genetic models and *in vitro* biochemical approaches to uncover the mechanisms of key regulatory cascades responsible for the onset of FOXG1 pathology at transcriptional and posttranscriptional levels. As congenital brain disorders can have a severe impact on human cognition and behavior, understanding the multifaceted pathways by which a single transcription factor, FOXG1, operates for the specification, differentiation and formation of the mature cerebral circuit will provide a fundamental step forward in the development of prospective clinical therapies. This is particularly feasible now that subtype-specific reprogramming technology is available. Continuing our effort to study this unique gene will help us establish the biological basis of the FOXG1 syndrome, as well as uncover the molecular logic underlying the sophisticated faculties of human cognition and behavior.

## Author Contributions

All authors made intellectual contributions to the manuscript and wrote the article.

## Conflict of Interest

The authors declare that the research was conducted in the absence of any commercial or financial relationships that could be construed as a potential conflict of interest.

## Footnotes

^1^http://developingmouse.brain-map.org/
